# Protective Nature of Mangiferin on Oxidative Stress and Antioxidant Status in Tissues of Streptozotocin-Induced Diabetic Rats

**DOI:** 10.1155/2013/750109

**Published:** 2013-09-12

**Authors:** Periyar Selvam Sellamuthu, Palanisamy Arulselvan, Subban Kamalraj, Sharida Fakurazi, Murugesan Kandasamy

**Affiliations:** ^1^Lab No. 102, Centre for Advanced Studies in Botany, University of Madras, Chennai, Tamil Nadu 600025, India; ^2^Department of Food Process Engineering, School of Bio-Engineering, SRM University, Kattankulathur, Tamil Nadu 603203, India; ^3^Laboratory of Vaccines and Immunotherapeutics, Institute of Bioscience, Universiti Putra Malaysia, 43400 Serdang, Selangor, Malaysia; ^4^Faculty of Medicine and Health Sciences, Department of Human Anatomy, Universiti Putra Malaysia, 43400 Serdang, Selangor, Malaysia

## Abstract

Oxidative stress plays an important role in the progression of diabetes complications. The aim of the present study was to investigate the beneficial effect of oral administration of mangiferin in streptozotocin (STZ)-induced diabetic rats by measuring the oxidative indicators in liver and kidney as well as the ameliorative properties. Administration of mangiferin to diabetic rats significantly decreased blood glucose and increased plasma insulin levels. The activities of antioxidant enzymes superoxide dismutase (SOD), catalase (CAT), and glutathione peroxidase (GPx) and level of reduced glutathione (GSH) were significantly (*P* < 0.05) decreased while increases in the levels of lipidperoxidation (LPO) markers were observed in liver and kidney tissues of diabetic control rats as compared to normal control rats. Oral treatment with mangiferin (40 mg/kg b.wt/day) for a period of 30 days showed significant ameliorative effects on all the biochemical and oxidative parameters studied. Diabetic rats treated with mangiferin restored almost normal architecture of liver and kidney tissues, which was confirmed by histopathological examination. These results indicated that mangiferin has potential ameliorative effects in addition to its antidiabetic effect in experimentally induced diabetic rats.

## 1. Introduction 

Diabetes mellitus (DM) is a series of endocrine metabolic disorders characterized by increased fasting and postprandial glucose levels as well as an insulin deficiency and/or defects of insulin action on regulation of glucose. More than 300 million people are expected to suffer from diabetes during the year 2025, and the global cost of managing diabetes and its various complications almost reaches one trillion US dollar annually as per worldwide projected epidemiology data [1, 2]. 

Reactive oxygen species (ROS) play a crucial role in the pathogenesis of some serious diseases/disorders, such as cancer, liver cirrhosis, cardiovascular diseases, diabetes, and inflammation associated malfunction [3]. These effects especially increased production of free radicals and its effect during diabetes are devastating and well documented [4, 5]. Streptozotocin (STZ) is frequently used to induce diabetes mellitus in experimental animal systems, and its toxic effects are produced by nitric oxide on pancreatic *β*-cells. The cytotoxic action of STZ is associated with the generation of ROS causing oxidative tissue damage [6].

Oxidative stress has been reported to play an essential role in diabetes right from its genesis to the development of microvascular and macrovascular complications. It results from an imbalance between the production and neutralization of ROS such as highly reactive hydroxyl radicals, superoxide anion, peroxyl radicals, singlet oxygen, peroxynitrite, and hydrogen peroxide. There is considerable evidence on altered antioxidant defenses including nonenzymatic and enzymatic antioxidant system, and the role of free radicals in the etiology of diabetes [7, 8]. The role of enzymatic and nonenzymatic antioxidants in the prevention/treatment of diabetes and its associated complications has also been reported [9].

Herbal remedies are apparently efficient, produce the least or no harmful effects in clinical experience, and are comparatively of low costs as compared to oral synthetic antidiabetic agents [10, 11]. Over the past years, variety of medicinal plants and their bioactive extracts have been reported to be effective in the cure and management of diabetes [12, 13]. Moreover, after the approbations made by World Health Organization on diabetes mellitus [14], investigation on effective antidiabetic agents from natural sources has become more significant. 

Antioxidants derived from medicinal plant source are gaining more attention as free radical scavengers as they protect against ROS-induced oxidative stress/damage. Nowadays, natural therapies gain importance as they have been shown to regulate the oxidative complications of diabetes [15, 16]. Antioxidants are used as well-known supportive therapy in the management/treatment of diabetes. Thus, there is an increasing demand for natural products with antidiabetic and antioxidant properties to attenuate induced oxidative stress and its complications [17]. Hence, it is strongly recommended that treatment with natural antioxidants may be a useful approach to management of diabetes and its complications. Therefore, the present investigation was aimed to evaluate the protective nature of mangiferin isolated from *Salacia chinensis* (*S. chinensis*) on oxidative stress and antioxidant defense system in chemically induced diabetic model. 

Mangiferin is a xanthonoid, a chemical compound present as a principal constituent of *Salacia *species [18]. The root of *S. chinensis* Linn is used in indigenous system of medicine, and it contains the major bioactive compound mangiferin [19]. Mangiferin is a natural polyphenol distribution in the Angiosperms occurring sporadically in some 13 dicot and 6 monocot families. Mangiferin reveals a broad range of pharmacological effects, including antidiabetic [20–22], antitumor [23], antiviral [24], immunomodulatory [25], antimicrobial, and antioxidant activities [26]. In Cuba, mangiferin is used traditionally to treat inflammatory conditions with its analgesic action and acts as an antioxidant under the brand name of Vimang. It is also available in the brand of Salaretin in Sri Lanka, used to treat diabetes. Mangiferins reveal excellent antioxidant and antiapoptotic properties, supporting their clinical application as trial neuroprotectors in pathologies concerning excitotoxic neuronal death [27]. 

The present study investigated the protective effect of oral administration of mangiferin isolated from *S. chinensis* on the vital tissues of STZ-induced diabetic rats with reference to oxidative stress indices and antioxidant defense system, with an observation to determine the possible mechanism of the antidiabetogenic action of mangiferin. The beneficial effect of mangiferin was compared with glibenclamide, a well known oral antidiabetic agent.

## 2. Material and Methods

### 2.1. Chemicals

Streptozotocin was procured from Sigma Chemical Co., St. Louis, MO, U.S.A. Radioimmunoassay kit for plasma insulin assay was purchased from Linco Research Inc., U.S.A. All other chemicals used were analytical grade and obtained from standard commercial suppliers.

### 2.2. Plant Material

The *S. chinensis* mature roots were harvested from Veenangaputtu, Karumpakkam, Thangal and Kurumpuram, Puducherry, and India. The plant material was identified by a taxonomist, and the same has been deposited in Centre for Advanced Studies in Botany (voucher specimen no. 778), University of Madras, Chennai, India.

### 2.3. Isolation of Mangiferin

The isolation and identification of mangiferin were carried out according to the standardized protocol from our previous studies [19]. Briefly, the isolation of mangiferin was done using various solvent systems by column chromatography, and the compound purity was confirmed through high performance liquid chromatography. The purity of mangiferin was confirmed with an authentic sample, which was procured from Sigma Aldrich (St. Louis, MO, U.S.A.). The purity of isolated mangiferin was closely resembled with authentic purity (Data not shown).

### 2.4. Animals

Male albino Wistar rats, weighing between 150 and 180 g, were obtained from TamilNadu Veterinary and Animal Sciences University, Chennai, India. The rats were maintained in an animal house with standard facilities. The animals were housed in clean cages and maintained at 25 ± 2°C under 12 h light-dark cycle, and they were fed with standard feed from Hindustan Lever Ltd., Bangalore, India. All the pharmacological experiments protocols were conducted according to the ethical norms approved by the Ministry of Social Justices and Empowerment, Government of India, and the Institutional Animal Ethics Committee Guidelines (IAEC no. 02/004/06).

### 2.5. Experimental Induction of Diabetes

The fasted rats were injected by a single intraperitoneal injection of a freshly prepared solution of STZ (55 mg/kg body weight) in 0.1 M cold citrate buffer (pH 4.5), and control rats were injected with citrate buffer alone. The rats were allowed to drink 5% glucose solution overnight to overcome the drug-induced hypoglycemia. One week after STZ administration, the animals with fasting glucose concentrations of over 250 mg/dL were considered to be diabetic and were used in the experiment. The treatment was started on the 8th day after STZ injection, and this was considered as the 1st day of treatment, and the same was continued for 30 days.

### 2.6. Experimental Design

The experimental animals were divided into four groups, each comprising of six animals as detailed in the following.

 Group 1: control rats receiving 0.1 M citrate buffer (pH 4.5).
 Group 2: diabetic controls.
 Group 3: diabetic rats given mangiferin (40 mg/kg b.wt/day) in aqueous solution orally for 30 days.
 Group  4: diabetic rats given glibenclamide (0.6 mg/kg b.wt/day) in aqueous solution orally for 30 days.

At the end of the total experimental period, the animals were fasted over night, anaesthetized and sacrificed, by cervical dislocation. The blood was collected with and/or without EDTA for plasma or serum separation, respectively, for further analysis. Plasma was used for the estimation of glucose by O-Toluidine method of Sasaki et al. [28]. The insulin concentration was determined by using radioimmunoassay kit according to the standard protocol. 

### 2.7. Preparation of Tissue Homogenate

The liver and kidney tissues from the control and experimental groups of rats were excised and rinsed with ice-cold saline. The preparation of tissue homogenates was done by a known amount of the liver and kidney tissues, homogenized in 0.1 M Tris-HCl buffer, pH 7.4 at 4°C, in a Potter-Elvehjem homogenizer with a Teflon pestle at 600 ×g for 3 min. The homogenates were centrifuged at 3,000 ×g for 10 min at 4°C using Sorvall refrigerated centrifuge. The supernatant was collected as tissue homogenate, and the same was used for the biochemical estimations. The protein concentration in the tissue homogenate was measured by the method of Lowry et al. [29]. 

### 2.8. Estimation of Biochemical Markers

Tissue homogenates were used for the following estimations. Thiobarbituric acids content was assayed by thiobarbituric acid reacting substances (TBARS) according to Ohkawa et al. [30]. Briefly, 0.2 mL of tissue homogenate, 0.2 mL of SDS, 1.5 mL of acetic acid, and 1.5 mL of TBA were added. The mixture was made up to 4 mL with water and then heated in a water bath at 95°C for 60 min. After cooling, 1 mL of water and 5 mL of n-butanol/pyridine mixture were added and shaken vigorously. After that it was centrifuged at 4000 rpm for 10 min; then, the organic layer was taken and measured at 532 nm absorbance. The 1,1′,3,3′-tetramethoxypropane was used as a standard. The level of lipid peroxides was expressed as mmoles of TBARS/100 g of tissues. 

The hydroperoxides in liver and kidney tissues were estimated by the method of Jiang et al. [31]. Estimation of hydroperoxides; 0.2 mL of tissue (liver and kidney) homogenates was treated with 1.8 mL of Fox reagent. (Eighty-eight mg butylated hydroxytoluene (BHT), 7.6 mg xylenol orange, and 9.8 mg ammonium sulphate were mixed to 90 mL of methanol and 10 mL 250 mM sulphuric acid.) And the reaction mixture was incubated at 37°C for 30 min. Finally, the reaction mixture was centrifuged, and the absorbance was read at 540 nm. Values were expressed as mmoles hydroperoxides/100 g tissues.

 Reduced glutathione was determined by the method of Sedlak and Lindsay [32]. 0.5 mL tissue homogenate was mixed with 0.2 M Tris buffer with pH of 8.2, and then contents were mixed with 0.1 mL of 0.01 M Ellman's reagent, (5,5′-dithiobis-(2-nitro-benzoic acid)) (DTNB), then centrifuged at 3000 g for 15 min. The absorbance was read at 412 nm. A series of standards treated in a similar way also run to determine the glutathione content. The amount of glutathione is expressed as mg/100 g of tissue.

 Superoxide dismutase was assayed following the method of Misra and Fridovich [33]. The tissue homogenate (0.1 mL) was mixed by reaction mixtures that contained sodium carbonate (1 mL, 50 mM), nitroblue tetrazolium (0.4 mL, 25 *μ*m), and hydroxylamine hydrochloride (0.2 mL, 0.1 mM). The samples were absorbed at 560 nm. 

 The activity of glutathione peroxidase was assayed by the method of Rotruck et al. [34]. The reaction mixture contained 0.2 mL of EDTA, 0.1 mL of sodium azide, 0.1 mL of H_2_O_2_, 0.2 mL of reduced glutathione, 0.4 mL of phosphate buffer and 0.2 mL tissue homogenate were incubated at 37°C for 10 min. The reaction was arrested by addition of 0.5 mL of TCA, and the tubes were centrifuged at 2000 rpm. To the supernatant, 3 mL of disodium hydrogen phosphate and 1.0 mL DTNB were added, and the colour developed was read at 420 nm immediately. The enzyme activity was expressed as *μ*moles of glutathione oxidized/min/mg of protein.

 The activity of catalase was assayed according to the method of Takahara et al. [35]. Phosphate buffer (1.2 mL) and 0.2 mL of tissue homogenate were mixed, and the reaction was started by the addition of 1.0 mL of H_2_O_2_ solution. Decrease in the absorbance was measured at 240 nm at 30 sec intervals for 3 min. For the enzyme blank, distilled water was used instead of hydrogen peroxide. The activity of enzyme was expressed as *μ*moles of H_2_O_2_ decomposed/min/mg of protein.

### 2.9. Histopathological Studies

Liver and kidney tissues for histopathological analysis were fixed in 10% buffered neutral formal saline solution. After fixation, tissues were embedded in paraffin; solid sections were cut at 4 *μ*m and stained with haematoxylin and eosin. The sections were examined under light microscope, and photomicrographs were taken.

### 2.10. Statistical Analysis

All the experimental data were statistically evaluated with SPSS/10 software and expressed as mean ± standard deviation for six rats in each group. Hypothesis testing methods included one way analysis of variance (ANOVA) followed by least significant difference (LSD) test. Values of *P* less than 0.05 were considered to point out statistical significance. 

## 3. Results

**Figure 1:**
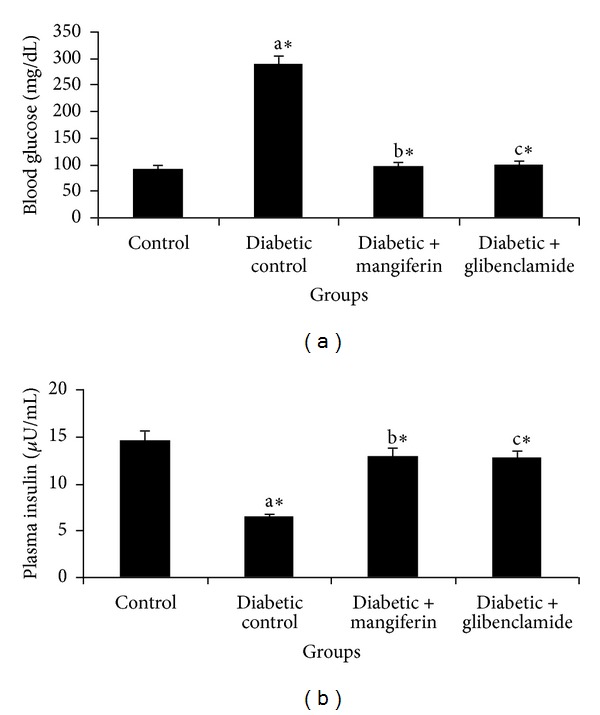
The levels of blood glucose and plasma insulin in control and experimental groups of rats. Data were given as mean ± standard deviation for six animals in each group. One way ANOVA is followed by *post hoc* test LSD. Values are statistically significant at *P < 0.05. ^a^Diabetic control rats were compared with control rats; ^b^mangiferin treated diabetic rats were compared with diabetic control rats; ^c^glibenclamide treated diabetic rats were compared with diabetic control rats.

Figures 1(a) and 1(b) demonstrate the levels of blood glucose and plasma insulin in the control and experimental groups of rats. There was a significant increase in the level of blood glucose and a concomitant decrease in the level of insulin in diabetic rats. Administration of mangiferin or glibenclamide to diabetic rats significantly decreased the level of blood glucose and increased the level of insulin.

**Figure 2:**
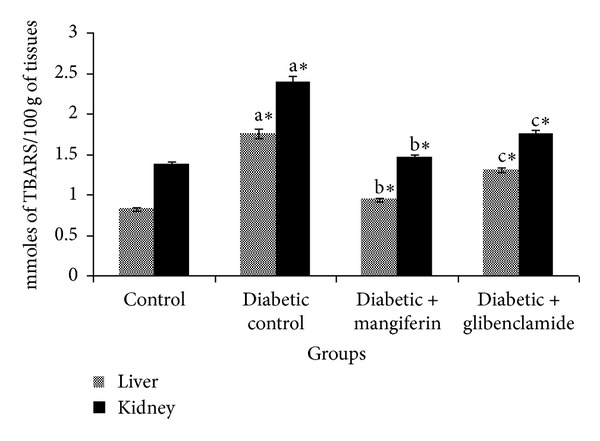
Levels of TBARS in liver and kidney of control and experimental groups of rats. Data were given as mean ± standard deviation for six animals in each group. One way ANOVA is followed by *post hoc* test LSD. Values are statistically significant at *P < 0.05. ^a^Diabetic control rats were compared with control rats; ^b^mangiferin treated diabetic rats were compared with diabetic control rats; ^c^glibenclamide treated diabetic rats were compared with diabetic control rats.

**Figure 3:**
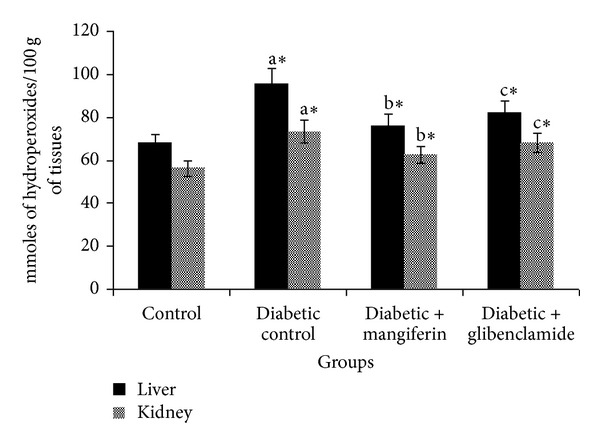
Levels of hydroperoxides in liver and kidney of control and experimental groups of rats. Data were given as mean ± standard deviation for six animals in each group. One way ANOVA is followed by *post hoc* test LSD. Values are statistically significant at *P < 0.05. ^a^Diabetic control rats were compared with control rats; ^b^mangiferin treated diabetic rats were compared with diabetic control rats; ^c^glibenclamide treated diabetic rats were compared with diabetic control rats.

The levels of TBARS and hydroperoxides in liver and kidney of control and experimental groups of rats are represented in Figures 2 and 3, respectively. The levels of TBARS and hydroperoxides were observed to be significantly increased in diabetic rats, when compared with control rats. The oral administration of mangiferin and glibenclamide treated diabetic rats significantly reduced the levels of TBARS and hydroperoxides to near normal in the liver and kidney tissues. 

**Figure 4:**
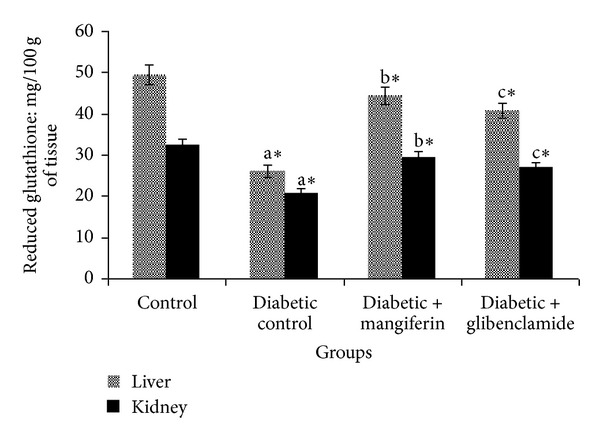
Levels of reduced glutathione (GSH) in liver and kidney of control and experimental groups of rats. Data were given as mean ± standard deviation for six animals in each group. One way ANOVA is followed by *post hoc* test LSD. Values are statistically significant at *P < 0.05. ^a^Diabetic control rats were compared with control rats; ^b^mangiferin treated diabetic rats were compared with diabetic control rats; ^c^glibenclamide treated diabetic rats were compared with diabetic control rats.

The concentrations of reduced glutathione (GSH) in liver and kidney of control and experimental groups of rats are shown in Figure 4. The levels of GSH were significantly decreased in liver and kidney of diabetic rats. These results were brought back to near normal, due to the oral administration of mangiferin and glibenclamide. 

**Figure 5:**
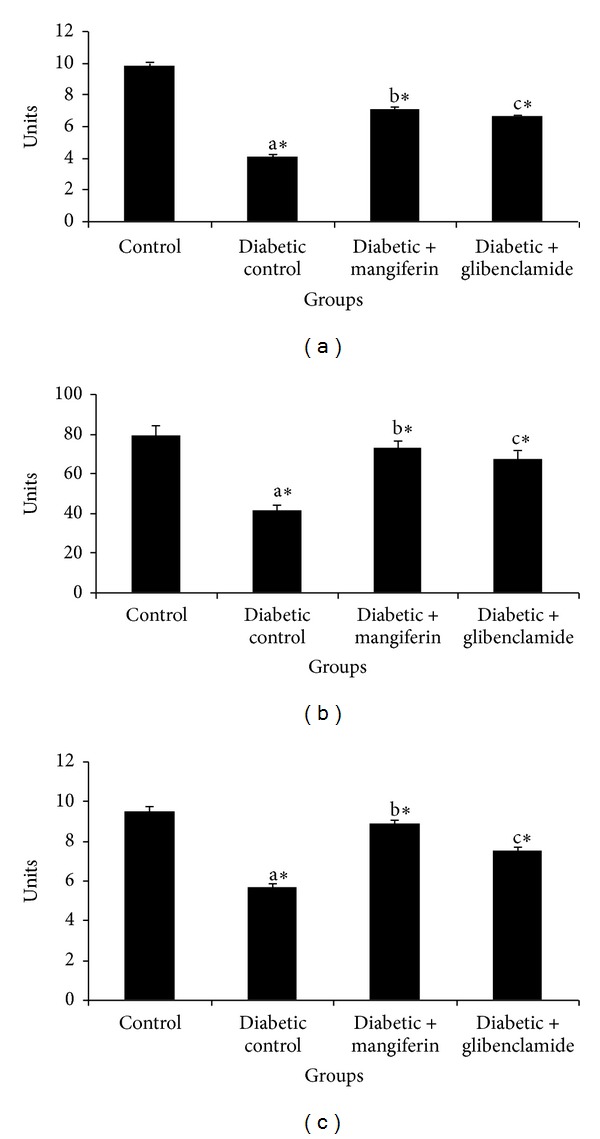
Activities of superoxide dismutase (SOD), catalase (CAT), and glutathione peroxidase (GPx) in liver and kidney of control and experimental groups of rats. Data were given as mean ± standard deviation for six animals in each group. One way ANOVA is followed by *post hoc* test LSD. Values are statistically significant at *P < 0.05. ^a^Diabetic control rats were compared with control rats; ^b^mangiferin treated diabetic rats were compared with diabetic control rats; ^c^glibenclamide treated diabetic rats were compared with diabetic control rats. The enzyme activities are expressed as SOD: 50% inhibition of epinephrine autooxidation/min; CAT: *μ*moles of H_2_O_2_ decomposed/min/mg of protein; GPx: *μ*moles of glutathione oxidized/min/mg of protein.

**Figure 6:**
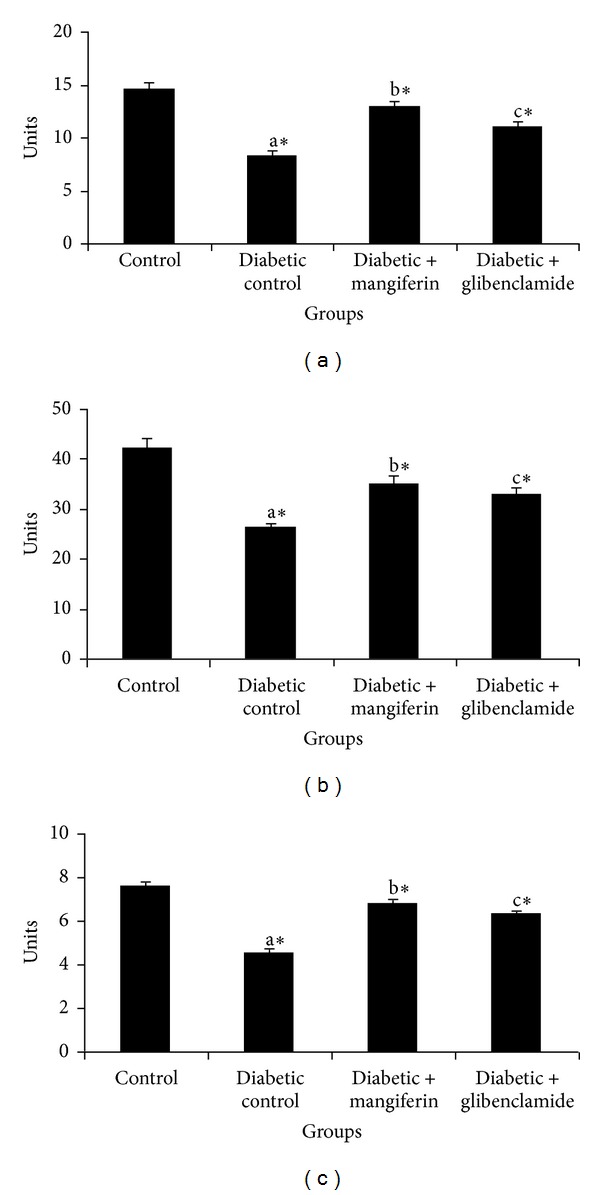
Activities of superoxide dismutase (SOD), catalase (CAT), and glutathione peroxidase (GPx) in kidney and of control and experimental groups of rats. Data were given as mean ± standard deviation for six animals in each group. One way ANOVA is followed by *post hoc* test LSD. Values are statistically significant at *P < 0.05. ^a^Diabetic control rats were compared with control rats; ^b^mangiferin treated diabetic rats were compared with diabetic control rats; ^c^glibenclamide treated diabetic rats were compared with diabetic control rats. The enzyme activities are expressed as SOD: 50% inhibition of epinephrine autooxidation/min; CAT: *μ*moles of H_2_O_2_ decomposed/min/mg of protein; GPx: *μ*moles of glutathione oxidized/min/mg of protein.

The activities of enzymatic antioxidants such as superoxide dismutase (SOD), catalase (CAT), and glutathione peroxidase (GPx) in the liver and kidney of control and experimental groups of rats are represented in Figures 5 and 6, respectively. The activities of SOD, CAT and GPx were significantly decreased in the liver and kidney of STZ-induced diabetic rats. The activities were brought back to normalcy in the STZ-induced diabetic rats due to the treatment with mangiferin and glibenclamide. These alterations were significantly regulated by mangiferin and glibenclamide treatment.

**Figure 7:**
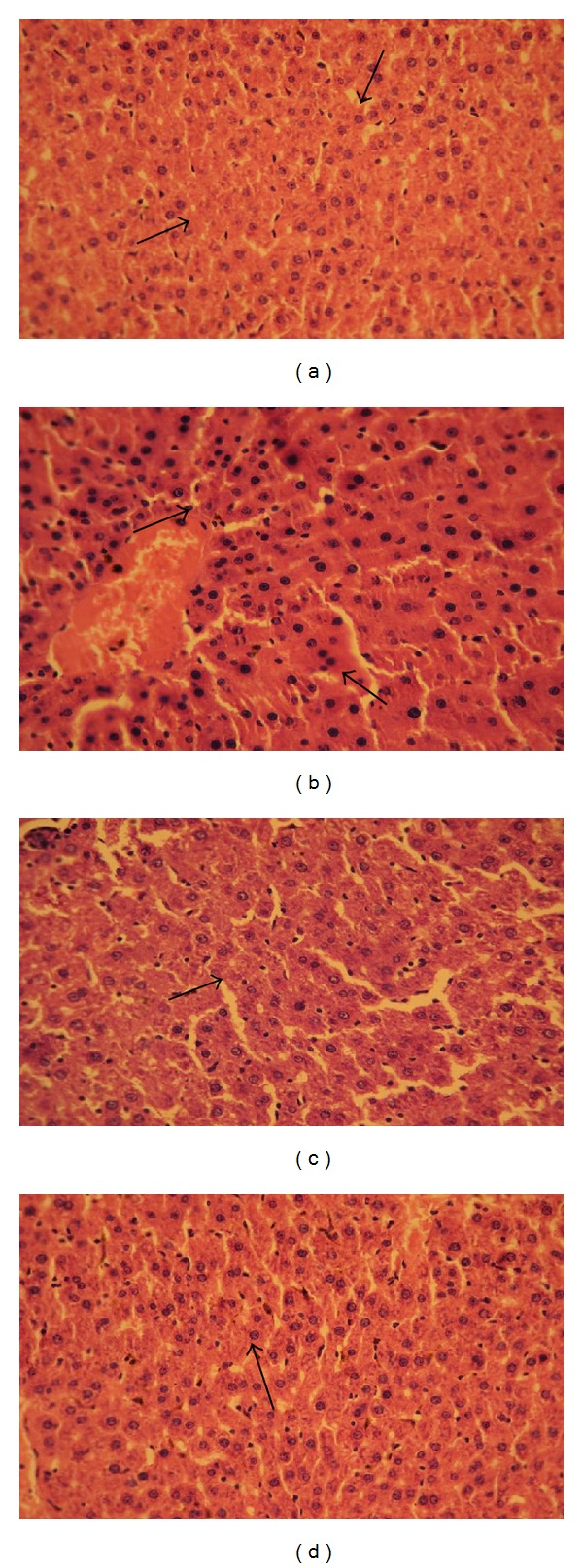
Histological observations in the liver tissue of control and experimental groups of rats. (a) Section of control rat liver tissue showing normal hepatocyte with vesicular nuclei; (b) section of diabetic rat liver tissue showing congested nuclei of the hepatocyte; (c) section of liver tissue from mangiferin treated diabetic rat showing near-normal hepatocyte with vesicular nuclei; (d) section of liver tissue from glibenclamide treated diabetic rat showing apparently normal architecture.

**Figure 8:**
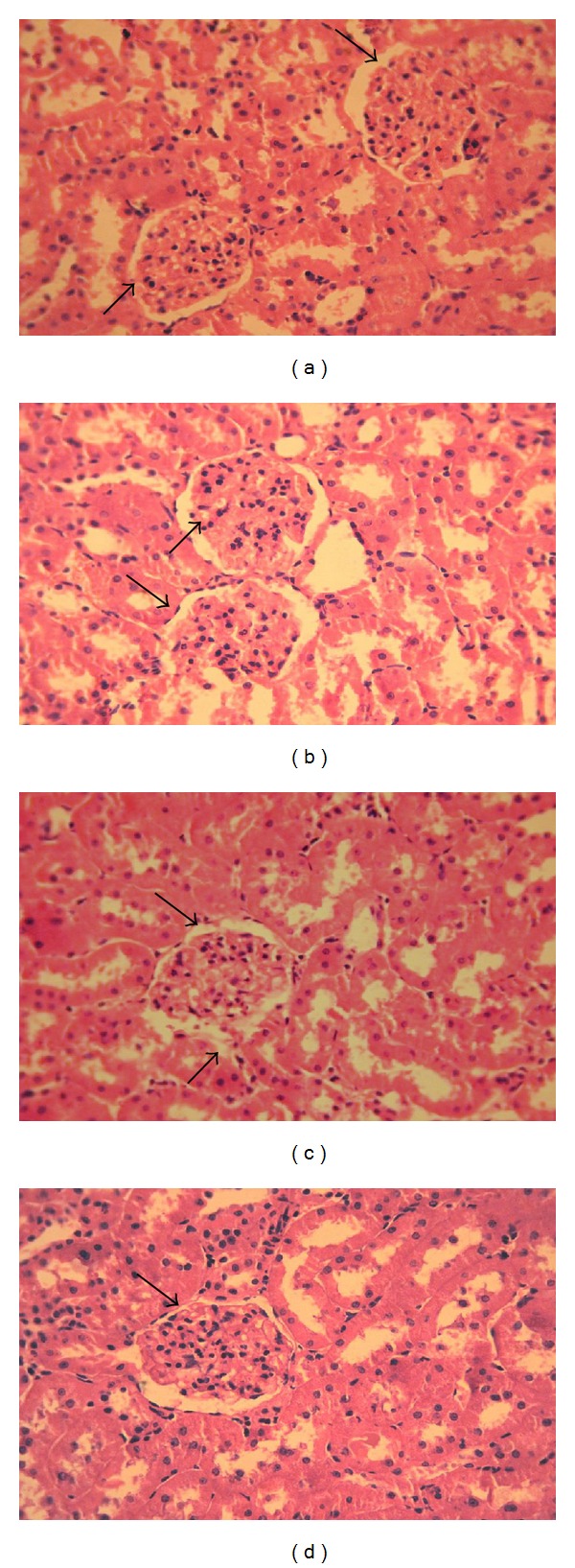
Histological observations in the kidney tissue of control and experimental groups of rats.(a) Section of control rat kidney tissue showing normal glomeruli and tubules; (b) section of diabetic rat kidney tissue showing thickening of vesicles and fibrosis in glomeruli; (c) section of kidney tissue from mangiferin treated diabetic rat showing near normal glomeruli and tubules; (d) section of kidney tissue from glibenclamide treated diabetic rat showing mild changes in glomeruli and tubules.

The histopathological observation revealed alterations in the liver and kidney of STZ-induced diabetic rats (Figures 7 and 8, resp.). The liver tissue of control rats showed a normal hepatocyte with vesicular nuclei (Figure 7(a)). The diabetic rat liver section represents congested nuclei of the hepatocyte and vacuolation hepatocyte nuclei (Figure 7(b)). The pathomorphological alteration observed in diabetic rats became apparently normal in their architecture with concentric arrangement of the hepatocytes with vesicular nuclei upon the treatment with mangiferin and glibenclamide (Figures 7(c) and 7(d), resp.).

The kidney tissue section of control rats represented a normal glomeruli and tubules (Figure 8(a)). The kidney tissue section of diabetic rats exhibited thickening of vesicles, fibrosis in glomeruli (Figure 8(b)). The mangiferin and glibenclamide treated diabetic rats represented near-normal glomeruli and tubules (Figures 8(c) and 8(d), resp.), when compared to diabetic control rats. 

## 4. Discussion

The free radical and reactive oxygen species are involved in various types of disorders/diseases including diabetes. The free radicals might be playing an important role in causation and complications in diabetes mellitus [36]. Antioxidant enzymes as well as nonenzymatic antioxidants are from the first line of defense against ROS induced oxidative damage in a living organism. 

Vital tissues are capable for antioxidant defense mechanisms, which include the concerted action of both antioxidant enzymes and nonenzymatic antioxidants. Activities of altered antioxidant enzymes SOD, CAT, GPx, and glutathione metabolism results in an imbalance of oxidant/antioxidant defense systems leading to the accumulation of highly reactive oxygen free radicals [37]. In the present study, we observed a decrease in antioxidant enzymes with elevation of lipid peroxidation markers in liver and kidney tissues of diabetic rats in addition to increased blood glucose with decreased plasma insulin levels.

The chronic hyperglycemia can cause oxidative stress, which leads to the cellular tissue damage. The structures and functions of an organ can be disturbed, during uncontrolled hyperglycemia. The STZ has cytotoxic effects against various vital tissues of pancreas, liver, and kidney. The STZ-induced diabetes is associated with the generation of ROS, which causes oxidative damage [38]. The mangiferin and glibenclamide treated diabetic rats showed the prevention of liver and kidney tissue dysfunction. This finding suggested that mangiferin might protect the liver and kidney tissues against the cytotoxic action of STZ. 

The lipid peroxidation and antioxidant potential have been measured in liver and kidney tissues of control and experimental groups of rats. The tissue lipid peroxidation in diabetic rats was increased, which might be due to an increase in the level of blood glucose [39]. Lipid peroxidation mediated tissue damage has been detected during the progress of diabetes mellitus; this is one of the specific features of chronic diabetes. The lipid radical and peroxide are risky to the body cells and allied with tissue damage. The level of lipid peroxidation was increased in the tissues of diabetic rats, which might be due to a significant increase in the levels of TBARS and hydroperoxides in the liver and kidney [40]. The most popularly used method to assay lipid peroxidation and free radical activity in biological sample is TBARS [41]. In the present study, it was also observed that the level of TBARS in liver and kidney tissues of STZ-induced diabetes was significantly increased when compared to the control. The oral treatment with mangiferin reduced the lipid peroxides near-normal levels in liver and kidney tissues of STZ-induced diabetic rats.

The hydroperoxides are highly toxic potential molecules, and they have the ability to destroy the defense enzymes and cell membrane. During the diabetic condition, the level of hydroperoxides was increased in plasma and tissues that leads to decreased activities of antioxidant enzymes. This is a favorable condition for the unlimited free radicals generation and consequent generation of lipid hydroperoxides [42]. The treatment of mangiferin and glibenclamide was significant decreased the hydroperoxides production in the liver and kidney tissues of diabetic rats. These observations lead us to conclude that mangiferin possessed antioxidant and antilipid peroxidant nature.

Glutathione protected the cellular system against toxic effects of lipid peroxidant. The level of reduced glutathione was decreased in diabetes condition [43]. Reduced glutathione (GSH) acts as a free radical scavenger and is involved in the repair of radical caused biological damage, and the decrease in the GSH content may alter antioxidant enzymes. Glutathione-S-transferase (GST) activity was reduced due to the reduction of GSH because it acts as a substrate for the activity of GST [44]. The decrease in the level of GSH in tissues represents increases in the utilization due to oxidative stress induced by STZ. In the present study, a significant decrease in GSH level in hepatic and renal tissues in STZ-induced diabetic rats. The mangiferin treated STZ-induced diabetic rats increased the level of GSH in liver and kidney tissues. These results suggested that the compound might increase the biosynthesis of GSH or reduce the oxidative stress or both. 

 The superoxide dismutase (SOD) and catalase (CAT) are two key scavenging enzymes that eliminate the toxic free radicals induced by STZ. The activities of SOD and CAT were reduced in liver and kidney tissues of diabetic rats [45]. SOD converts the superoxide radicals into H_2_O_2_ and molecular oxygen. CAT protected the tissues from highly reactive hydroxyl radical through catalyzing the reduction of hydrogen peroxides [46]. The decrease of SOD and CAT activities might result from the inactivation by glycation of the enzyme [47]. The most effective defense mechanism against diseases is the removal of O_2_^−^ and OH. The mangiferin treated STZ-induced diabetic rats (liver and kidney) showed an increase in the activities of SOD and CAT to near-normal. These results revealed that mangiferin may contain a free radical scavenging activity and prevent pathological alteration caused by O_2_^−^ and OH^−^.

 In diabetes, the activities of CAT and GPx are significantly decreased by superoxide radical and by glycation reactions. The glutathione peroxidase along with glutathione catalyzed the reduction of hydrogen peroxide into nontoxic metabolites. During diabetes, there is a decrease in the concentration of GSH that reduced the activities of GPx [48]. Glutathione, a tripeptide functions as a scavenger of free radicals formation and also an essential cosubstrate for GPx. As NADPH necessary for GSH regeneration was utilized by the polyol pathway which is prominent in chronic hyperglycemic conditions, there occurs a depletion of GSH resulting in lowered GPx activity [49]. Reduced activities of enzymatic antioxidants have been observed during diabetes, and this may result in a number of deleterious effects due to the accumulation of free radicals [46]. The activities of tissue (liver and kidney) GPx antioxidant enzyme were normalized by the treatment with mangiferin in STZ-induced diabetic rats. The activities of these antioxidant enzymes influence the susceptibility of various tissues especially liver and kidney to oxidative stress. In the present study, we also observed a significant decrease in the activities of antioxidant enzymes in liver and kidney tissues of diabetic rats when compared to normal control animals. These findings concluded that mangiferin acts as an antioxidant agent against STZ-induced diabetes.

 The structural damage of the tissues might be due to excessive production of free radicals and oxidative stress; such stress may also play an important role in the development of STZ, induced experimental diabetes [40, 50]. Various reports showed that natural antioxidants from plant sources have been suggested to have beneficial effects in the treatment of oxidative stress disease [16, 51]. In this respect, the protective effect of the mangiferin on streptozotocin related cytotoxicity was examined in the liver and kidney tissues of rats exposed to STZ. The liver is an organ of central metabolic importance and is known to undergo oxygen free radicals mediated injury in diabetes mellitus [52]. After the oral administration of mangiferin, the liver injury in STZ-induced diabetic rats was reduced when compared to untreated STZ-induced diabetic rats. It was concluded that mangiferin has a protective effect against the hepatotoxicity produced by STZ. Moreover, diabetic kidney exhibits a characteristic pattern of changes, eventually leading to renal insufficiency or complete kidney failure due to oxidative damage induced by STZ. The decrease of the degenerative changes in the diabetic group given mangiferin indicated that this phytochemical prevented the damage in the kidney tissue of diabetic rats. This investigation suggests that mangiferin has a strong tissue protective nature against chemically induced diabetic experimental model. 

 In conclusion, our findings showed that mangiferin markedly reduced hyperglycemia and associated oxidative complications in STZ-induced diabetic rats, decreased glucose level, and increased antioxidants markers including enzymatic and nonenzymatic antioxidants. Thus, the present study has shown that mangiferin has a liver and kidney protective nature against STZ-induced diabetic experimental rats due to decreasing the levels of oxidative markers and improvement of antioxidants systems. Further detailed investigations are in progress to elucidate the exact mechanism by which mangiferin elicits its modulatory properties.
